# SOCS3 Suppression Is Associated with Hippocampal Inflammation in a Mouse Model of Intermittent Hypoxia

**DOI:** 10.3390/biomedicines14102373

**Published:** 2026-10-18

**Authors:** Yu Shi, Yelei Han, Junxiu Song, Zhan Zhao, Yan Wang, Jing Zhang, Guangsheng Li, Congcong Wang, Qiling Cai, Yubao Wang

**Affiliations:** 1 Respiratory Department, Tianjin Medical University General Hospital, Tianjin Medical University, Tianjin 300070, China; shiyutj_0509@163.com (Y.S.); zhaozhan726@163.com (Z.Z.); wangy0504@126.com (Y.W.); tjzyyzhangjing@163.com (J.Z.); lgszyy@126.com (G.L.); 2 Respiratory Department, Chengdu Fifth People’s Hospital, Chengdu 611130, China; hanyelei1996@126.com; 3 Respiratory Department, Tianjin Haihe Hospital, Tianjin 300350, China; songjunxiu156@163.com; 4 NHC Key Laboratory of Hormones and Development, Chu Hsien-I Memorial Hospital and Tianjin Institute of Endocrinology, Tianjin Medical University, Tianjin 300134, China; 13002219612@163.com (C.W.); caiqiling1969@163.com (Q.C.); 5 Tianjin Key Laboratory of Metabolic Diseases, Tianjin Medical University, Tianjin 300134, China

**Keywords:** intermittent hypoxia, SOCS3, H3K27me3, neuroinflammation, cognitive impairment

## Abstract

**Background/Objectives:** Intermittent hypoxia (IH), a feature of obstructive sleep apnea (OSA), is associated with hippocampal inflammation and cognitive dysfunction. We examined SOCS3 expression and a possible MALAT1/EZH2/H3K27me3 regulatory relationship in a mouse IH model and BV2 cells. **Methods:** C57BL/6J mice were exposed to IH for 4 weeks, followed by Morris water maze testing and analysis of hippocampal tissue. BV2 cells were exposed to IH and used for transfection, RNA immunoprecipitation, and chromatin immunoprecipitation experiments. **Results:** In mice, IH was associated with poorer water maze performance, lower hippocampal SOCS3, and higher levels of inflammatory markers, MALAT1, and H3K27me3. Similar molecular changes were observed in BV2 cells. SOCS3 overexpression reduced TRAF6, total NF-κB p65, TNF-α, and IL-1β protein levels in BV2 cells. MALAT1 was enriched by EZH2 immunoprecipitation, H3K27me3 was enriched at the SOCS3 promoter, and MALAT1 knockdown reduced total H3K27me3 while increasing SOCS3 protein. **Conclusions:** These findings associate reduced SOCS3 with hippocampal inflammation after IH and support further investigation of MALAT1 and EZH2 in its regulation. The cellular source of the in vivo inflammatory markers and the causal role of EZH2 require direct testing.

## 1. Introduction

Obstructive sleep apnea (OSA) is a disease that is characterized by repeated partial or complete airway obstruction during sleep [1]. Due to the aging population and the continuing obesity epidemic, the prevalence of OSA is increasing each year [2]. Studies have shown that OSA patients have deficits in cognitive abilities related to attention, vigilance, memory and executive functions during the daytime test [3], which may be related to hippocampal damage [4]. Intermittent hypoxia (IH) is the main clinical feature of OSA and may lead to cognitive dysfunction [5]. Microglia are highly specialized tissue macrophages in the brain and have important functions in neuronal development, homeostasis, and immune responses [6]. Hypoxia can cause microglia to release inflammatory factors to promote neuronal cell damage, and prolonged exposure to hypoxia can cause neuronal cell loss and death [7]. However, the mechanisms of hippocampal inflammation in response to IH are still unclear. Therefore, we examined hippocampal inflammatory markers in mice and the response of BV2 cells to IH. The effects of the proposed pathway on clinical cognition remain to be established.

Suppressor of cytokine signaling (SOCS) proteins are induced by intracellular cytokines and can inhibit the signal transduction of cytokines in the central nervous system and immune system cells [8]. Research has shown that SOCS3, which is a member of the SOCS family, plays an important role in the control mechanism of adaptive immunity and innate immunity to avoid overstimulation [9]. Toll-like receptor (TLR) activation is a major inducer of neuroinflammation. A study reported that under OSA conditions, TLR2 can induce microglial activation and initiate neuroinflammation in the central nervous system [10]. Tumor necrosis factor receptor-associated factor 6 (TRAF6) is a member of the tumor necrosis factor receptor family that can mediate the activation of nuclear factor (NF)-κB, which depends on the myeloid differentiation factor 88 (MyD88) and Mitogen-Activated Protein Kinase (MAPK) pathways [11]. SOCS3 can negatively regulate TLR-mediated microglial activation and the inflammatory immune response by degrading TRAF6 [12]. Therefore, we hypothesize that there may be abnormal SOCS3 expression associated with the cognitive impairment caused by OSA, and this abnormal expression has an effect through the TRAF6/NF-κB pathway.

Recent studies have shown that histone methylation can participate in the epigenetic control of the immune response [13]. Among all chromatin modifications, the specific methylation of histone lysine sites is a basic regulatory mechanism, and it has been shown that the trimethylation of histone H3 lysine 27 (H3K27me3) is related to gene repression [14]. Enhancer of zeste homolog 2 (EZH2) is the catalytic subunit of polycomb repressive complex 2 (PRC2) and can catalyze the methylation of H3K27 to generate H3K27me3 [15]. A study on Parkinson’s disease (PD) showed that long non-coding RNA (lncRNA) metastasis-associated lung adenocarcinoma transcript 1 (MALAT1) can inhibit the neuroprotective effect of nuclear factor (erythroid-derived 2)-like-2 factor (Nrf2) by recruiting EZH2 to the Nrf2 promoter, thereby promoting neuroinflammation [16]. Prior work has also linked microglial EZH2 activity to repression of SOCS3 [12]. Together with reports of MALAT1 association with EZH2 [16], this provides a rationale to examine whether MALAT1, EZH2, and H3K27me3 are associated with lower SOCS3 after IH. We tested this hypothesis in whole hippocampal tissue and in BV2 cells, while recognizing that these systems cannot establish the same cell-specific mechanism in vivo.

## 2. Materials and Methods

### 2.1. Animals and Experimental Conditions

Twenty 11-week-old male wild-type C57BL/6J (C57) mice were purchased from GemPharmatech Co., Ltd. (Nanjing, China) and were housed under specific pathogen-free (SPF) conditions at the Animal Experiment Center of Tianjin Medical University. The mice were kept in an air-conditioned room with a constant temperature of 23 °C and a 12 h light/12 h dark cycle and were given sufficient water and food. After 1 week of adaptive feeding, C57 mice (weight 27–32 g, 12 weeks old) were randomly divided into a control group (*n* = 10) and an IH group (*n* = 10). We used Visual C++ to write a gas delivery control program to deliver specific gases to the exposure chamber to simulate IH during OSA exposure [17]. The specific exposure method involved reducing the ambient oxygen concentration to 5% during each IH exposure cycle for 30 s and then quickly restoring the oxygen concentration to 21% for 90 s, 8 h a day for a total of 4 weeks. At the end of the exposure period, the mice were anesthetized by an intraperitoneal injection of 10% chloral hydrate and then sacrificed. All animal experiments followed ARRIVE and NIH guidelines, and the protocol was approved by Tianjin Medical University’s Animal Ethics Committee (Ethics Approval No. IRB2024-DWFL-194, approval date: 23 February 2024).

### 2.2. Morris Water Maze

We assessed spatial learning and reference memory with a Morris water maze. During five training days, we recorded the latency to find the hidden platform. On day six, the platform was removed for a 120 s probe trial, and platform-site crossings and time in the target quadrant were recorded. Water temperature was approximately 24 °C, and trajectories were recorded with SMART v3.0.03 software.

### 2.3. Cell Culture

BV2 mouse microglial cells (BeNa Culture Collection, Beijing, China) were cultured in Dulbecco’s modified Eagle’s medium (DMEM, C11960500BT, Gibco, Waltham, MA, USA) supplemented with 10% fetal bovine serum (FBS) at 37 °C with 5% CO_2_ in a humidified incubator. Cells were seeded in six-well or 24-well plates. Upon reaching 70% confluence, cells were divided into the normal control (NC) group and intermittent hypoxia (IH) group. The IH group was exposed to cyclic IH stimulation for 8 h using a programmable hypoxia chamber equipped with a built-in oxygen sensor for real-time monitoring and automatic feedback regulation of ambient oxygen levels. A precision gas delivery system mixed high-purity nitrogen and oxygen to enable rapid switching between hypoxic and normoxic conditions. The cyclic paradigm consisted of 30 s of hypoxia (1.5% O_2_) followed by 60 s of reoxygenation (21% O_2_), with 5% CO_2_ maintained constantly and nitrogen used for gas balance throughout the intervention. Oxygen concentration was continuously recorded during exposure. The NC group was incubated under normoxic conditions for the same duration, with all culture conditions identical except for oxygen fluctuation.

### 2.4. Western Blotting (WB)

Proteins were extracted from cells and the frozen hippocampal tissues of mice. The proteins were further separated using 10% or 12.5% sodium dodecyl sulfate polyacrylamide gel electrophoresis (SDS-PAGE) and then transferred to nitrocellulose (NC) membranes by a wet protein transfer system (L00686C, GenScript, Nanjing, China). The membranes were incubated with Tris-buffered saline Tween (TBST) containing 5% skim milk for 1 h to avoid nonspecific binding. Then, the membranes were incubated overnight at 4 °C with primary antibodies against SOCS3 (1:1000, ab16030, Abcam, Cambridge, MA, USA), TRAF6 (1:4000, 66498-1-Ig, Proteintech, Rosemont, IL, USA), NF-κB-p65 (1:500, ab7970, Abcam, Cambridge, MA, USA), TNF-α (1:1000, ab183218, Abcam, Cambridge, MA, USA), interleukin (IL)-1β (1:1000, 16806-1-AP, Proteintech, Rosemont, IL, USA), β-actin (1:5000, AP0060, Bioworld, St. Louis Park, MN, USA), H3K27me3 (1:1000, ab192985, Abcam, Cambridge, MA, USA), and histone H3 (1:1000, 17168-1-AP, Proteintech, Rosemont, IL, USA). After being washed with TBST, the membranes were incubated with horseradish peroxidase (HRP)-conjugated goat anti-rabbit/mouse secondary antibodies (1:5000 dilution) for 1 h at room temperature. After being washed, the membranes were exposed to ECL solution, and the results were analyzed by ImageJ software (version 1.48v, National Institutes of Health, Bethesda, MD, USA) and normalized to β-actin or histone H3.

### 2.5. Real-Time Quantitative Polymerase Chain Reaction (qRT-PCR)

Hippocampal tissue and BV2 cells were lysed with TRIzol reagent, and then total RNA was extracted according to the manufacturer’s protocol. Then, a reverse transcription kit (AT301-03, TransGen Biotech, Beijing, China) was used to synthesize cDNA. qRT-PCR analysis was performed using a SYBR Green PCR kit. The 2-ΔΔCt method was used to calculate the expression relative to that of β-actin.

The primer sequences for MALAT1 were forward: 5′-CAGTGCTGGGTGGGAATGTA-3′ and reverse: 5′-TGGCCAAGTCTGTTATGTCCA-3′.

The primer sequences for β-actin were forward: 5′-CATTGCTGACAGGATGCAGAAGG-3′ and reverse: 5′-TGCTGGAAGGTGGACAGTGAGG-3′.

### 2.6. Immunohistochemistry (IHC)

Paraffin sections of mouse brain tissue were deparaffinized with xylene, hydrated with ethanol, and washed 3 times with phosphate-buffered saline (PBS). Then, the slides were placed in 0.01 M citrate buffer, heated to boiling in a microwave oven, and cooled to 60 °C naturally, after which the process was repeated once. The cells were incubated with a 0.03% hydrogen peroxide-containing methanol solution for 20 min at room temperature and washed 3 times with PBS. Then, the slides were blocked with 1% BSA for 1 h. After being washed again, the tissue sections were incubated with SOCS3 (1:200, ab16030, Abcam), H3K27me3 (1:200, ab192985, Abcam), TNF-α (1:200, ab183218, Abcam), and IL-1β (1:200, 16806-1-AP, Proteintech) primary antibodies diluted with 1% BSA at 4 °C overnight. The next day, the sections were washed 3 times with PBS and then incubated for 1 h at room temperature with HRP-conjugated goat anti-rabbit/mouse secondary antibodies diluted 1:200. After being washed with PBS, 3,30-diaminobenzylamine (DAB, DA1010, Solarbio, Beijing, China) was added dropwise for color development until the sections appeared light brown. Finally, hematoxylin was used to stain the nuclei, and the slides were dehydrated and fixed for photographic observation.

### 2.7. Immunofluorescent (IF) Labeling

The BV2 cell slides were fixed, permeabilized and blocked. Then, the slides were incubated with anti-SOCS3 (1:100, ab16030, Abcam) and anti-H3K27me3 (1:100, ab192985, Abcam) antibodies at 4 °C overnight. Next, fluorescein isothiocyanate (FITC)/tetramethylrhodamine isothiocyanate (TRITC)-labeled goat anti-rabbit secondary antibodies were added and incubated for 1 h at room temperature in the dark. The nuclei were stained with 4,6-diamidino-2-phenylindole (C0065, Solarbio, Beijing, China) for 60 s. A fluorescence microscope was used to observe and take images.

### 2.8. RNA-Binding Protein Immunoprecipitation (RIP) Assay

A Magna RIP kit (17-701, Millipore, Burlington, MA, USA) was used to examine the binding of MALAT1 and EZH2 in BV2 cells. Cells that reached 90% confluence were lysed in RIP lysis buffer, and then the supernatant was collected and divided into two equal aliquots. Then, the washed magnetic beads were incubated with 5 μL of anti-EZH2 (21800-1-AP, Proteintech) antibody or immunoglobulin G (IgG) and 100 μL of RIP washing buffer for 30 min at room temperature. The coupled magnetic beads were incubated with the cell lysate overnight at 4 °C to precipitate MALAT1. The immunoprecipitated RNA was purified, and qRT-PCR was used to measure the enriched MALAT1.

### 2.9. Chromatin Immunoprecipitation (ChIP) Assay

ChIP-qPCR analysis was used to evaluate the binding of H3K27me3 and the promoter region of the SOCS3 gene. A chromatin extraction kit (P-2001, Epigentek, Farmingdale, NY, USA) was used to extract chromatin from BV2 cells according to the manufacturer’s instructions. Then, the Magnetic ChIP Kit (P-2026, Epigentek, Farmingdale, NY, USA) was used to perform the ChIP assay. Chromatin was sheared using a sonicator and then incubated with anti-H3K27me3 antibodies (1:50, ab192985, Abcam) or negative control IgG and magnetic beads on a shaker for 2 h. The magnetic beads were separated, washed, and digested with proteinase K, and the purified DNA was analyzed by qRT-PCR.

Primers for the SOCS3 promoter were as follows: forward: 5′-CGCTTCGGGACTAGGTAGGA-3′ and reverse: 5′-AGAAACCGGGAAAAGCTCCC-3′. This amplicon targets the SOCS3 promoter region (mm10, chr11:117968804–117968888), with a product length of 85 bp.

### 2.10. Cell Transfection

We constructed a SOCS3 overexpression plasmid (EX-Mm01783-M37, GeneCopoeia, Rockville, MD, USA) and normal plasmids (EX-NEG-M37, GeneCopoeia, Rockville, MD, USA) of the same model were used as a control. MALAT1 siRNA and NC siRNA were purchased from GenePharma (Shanghai, China). The sequences of MALAT1 siRNA were as follows: sense: 5′-GCUUCCAGUUGAAUUGAAATT-3′; antisense: 5′-UUUCAAUUCAACUGGAAGCTT-3′. The sequences of NC siRNA were as follows: sense: 5′-UUCUCCGAACGUGUCACGUTT-3′; antisense: 5′-ACGUGACACGUUCGGAGAATT-3′. BV2 cells were inoculated into 6-well plates and cultured in a 37 °C in a 5% carbon dioxide incubator until they reached 70% confluence. Transfection reagent (IV1216150, Invitrogen, Waltham, MA, USA) was used to transfect BV2 cells with the SOCS3 overexpression plasmid, NC plasmid, MALAT1 siRNA, or NC siRNA according to the manufacturer’s protocol.

### 2.11. Statistical Analysis

Statistical analyses were carried out with SPSS version 19.0 (IBM Corporation, Armonk, NY, USA). All quantitative graphs were generated in GraphPad Prism 10.0 (GraphPad Software Inc., La Jolla, CA, USA). For comparisons between two experimental groups, the unpaired Student’s *t*-test was utilized. Differences across three or more groups were evaluated via one-way analysis of variance (ANOVA), followed by Bonferroni-corrected post hoc tests for pairwise comparisons. Repeated-measures ANOVA was applied for the escape-latency data acquired from the Morris water maze test to account for within-mouse longitudinal measurements. All quantitative results are presented as mean ± standard error of the mean (SEM). A two-tailed *p*-value less than 0.05 was defined as the threshold for statistical significance.

## 3. Results

### 3.1. IH Exposure Impaired Cognitive Function in C57 Mice

**Figure 1:**
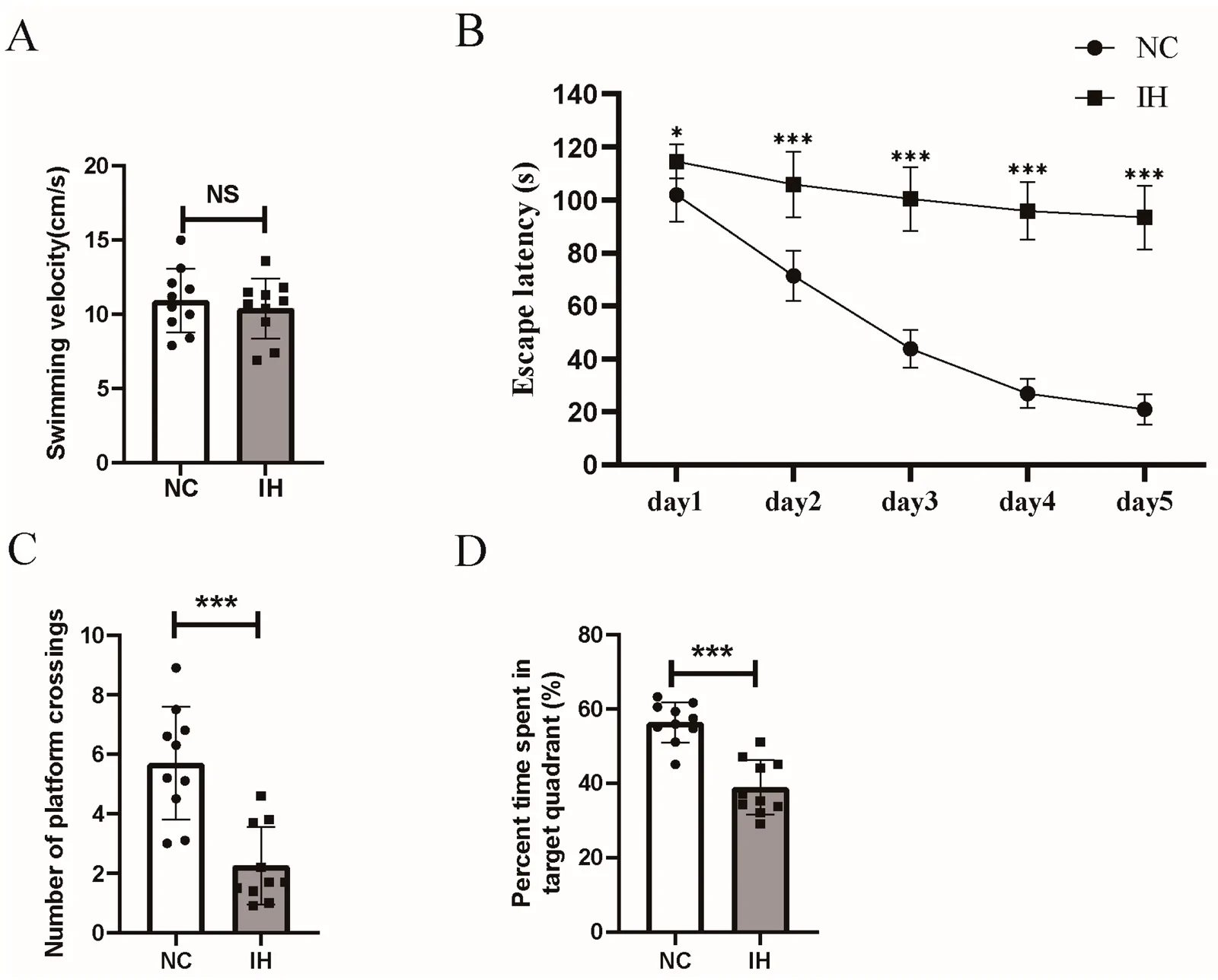
C57 mice subjected to intermittent hypoxia (IH) exhibit impaired spatial learning and memory. (**A**) Swimming speed in the Morris water maze was detected to assess motor function. (**B**) Escape latency across the first five training days reflects spatial learning capacity, analyzed by repeated-measures ANOVA. (**C**) Number of platform crossings and (**D**) percentage of time spent in the target quadrant were applied to assess spatial-memory performance. Data are shown as mean ± SEM, *n* = 10 mice per group. * *p* < 0.05, *** *p* < 0.001, ns, not significant. Panels A, C and D were analyzed using an unpaired Student’s *t*-test; escape-latency data in panel B were analyzed by repeated-measures ANOVA.

There was no difference in the swimming speed between the two groups of mice in the Morris water maze, indicating that IH exposure did not affect motor abilities (Figure 1A). The escape latency of mice exposed to IH was significantly longer than that of mice in the control group, and this difference became particularly obvious beginning on the second day (Figure 1B). These results are consistent with impaired performance on the spatial learning task after IH. Mice exposed to IH crossed the platform significantly fewer times than mice in the control group during the probe test (Figure 1C). In addition, mice exposed to IH spent significantly less time in the target quadrant during the probe test than mice in the control group (Figure 1D). These results indicate poorer performance on the spatial memory probe trial after IH.

### 3.2. The Expression of Inflammatory Factors Was Increased in the Hippocampus of C57 Mice Exposed to IH, While the Expression of SOCS3 Was Decreased

**Figure 2:**
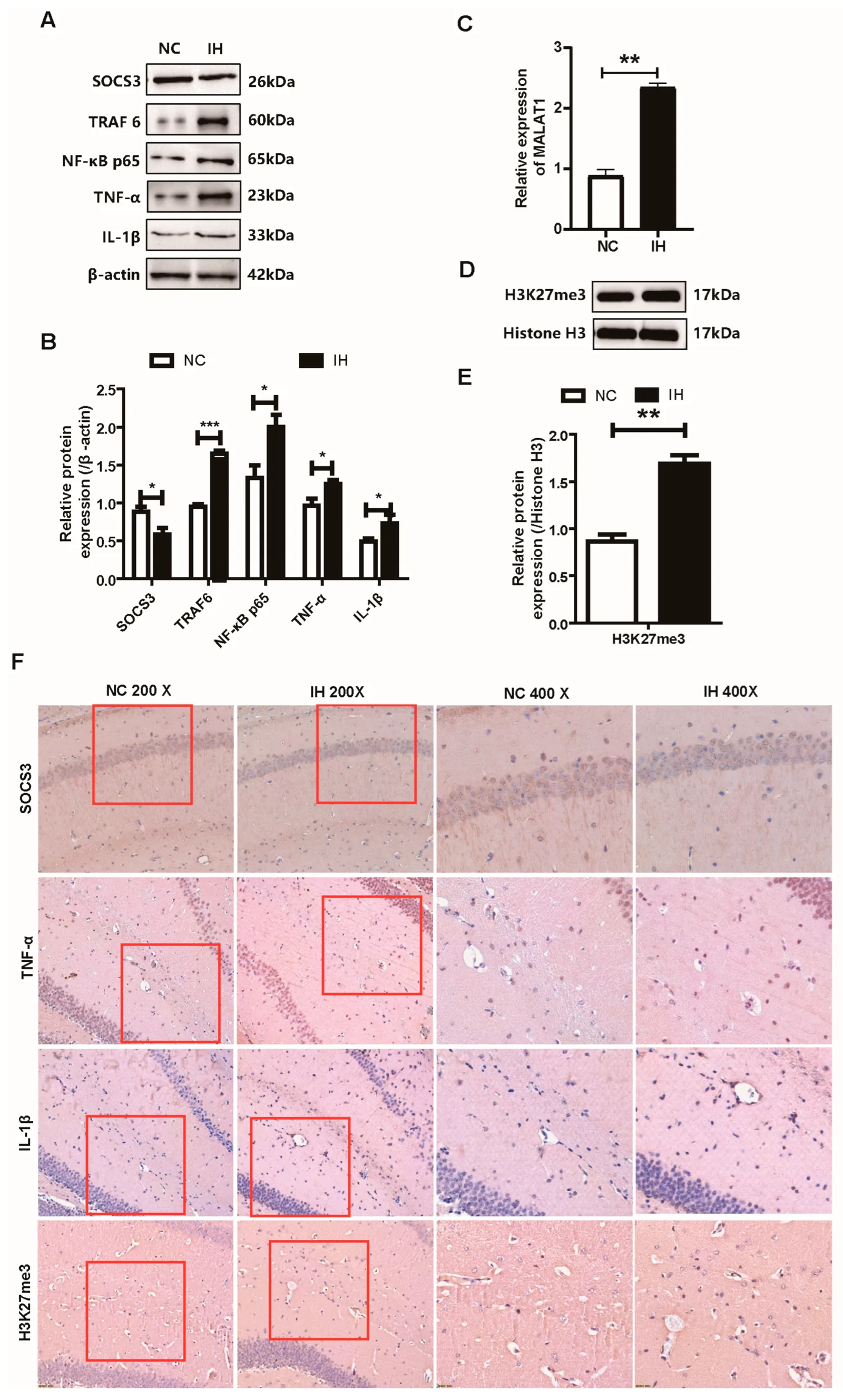
Changes in the expression of SOCS3 and related factors in the hippocampus of C57 mice exposed to IH. (**A**,**B**) Western blot for SOCS3, TRAF6, NF-κB-p65, TNF-α, IL-1β (normalized to β-actin, *n* = 4 per group). (**C**) qRT-PCR analysis of MALAT1 (*n* = 3 per group). (**D**,**E**) Western blot of H3K27me3 normalized to histone H3 (*n* = 3 per group). (**F**) Immunohistochemistry for TNF-α, IL-1β, SOCS3 and H3K27me3; red boxes mark representative positive cells are shown (*n* = 3 per group). * *p* < 0.05, ** *p* < 0.01, *** *p* < 0.001, unpaired Student’s *t*-test.

We measured the expression of SOCS3, TRAF6, NF-κB-p65, TNF-α, and IL-1β in the hippocampus of the two groups of mice by WB (Figure 2A,B). The results showed that the expression of TNF-α and IL-1β in the IH group was significantly higher than that in the control group. This result suggests that IH exposure can induce inflammation in the hippocampus of C57 mice. In addition, we also found that the expression of SOCS3 in the hippocampus of mice exposed to IH was significantly reduced, while the expression of TRAF6 and NF-κB-p65 was increased. We analyzed the expression of MALAT1 in the hippocampus in the two groups of mice by qRT-PCR and found that MALAT1 expression was higher in the IH group (Figure 2C). Next, we measured the expression of H3K27me3 relative to histone H3 in the hippocampus in the two groups of mice by WB and found that the trimethylation level of H3K27 in the IH group was higher (Figure 2D,E). Finally, we verified the changes in the expression of the inflammatory factors TNF-α, IL-1β, SOCS3 and H3K27me3 in the hippocampus in the two groups of mice by IHC (Figure 2F). The results showed that the expression of TNF-α, IL-1β and H3K27me3 was increased in the hippocampus in the IH group, while the expression of SOCS3 was decreased, which was consistent with the Western blot results.

### 3.3. The Expression of Inflammatory Factors Was Increased in BV2 Cells Exposed to IH, While the Expression of SOCS3 Was Decreased

**Figure 3:**
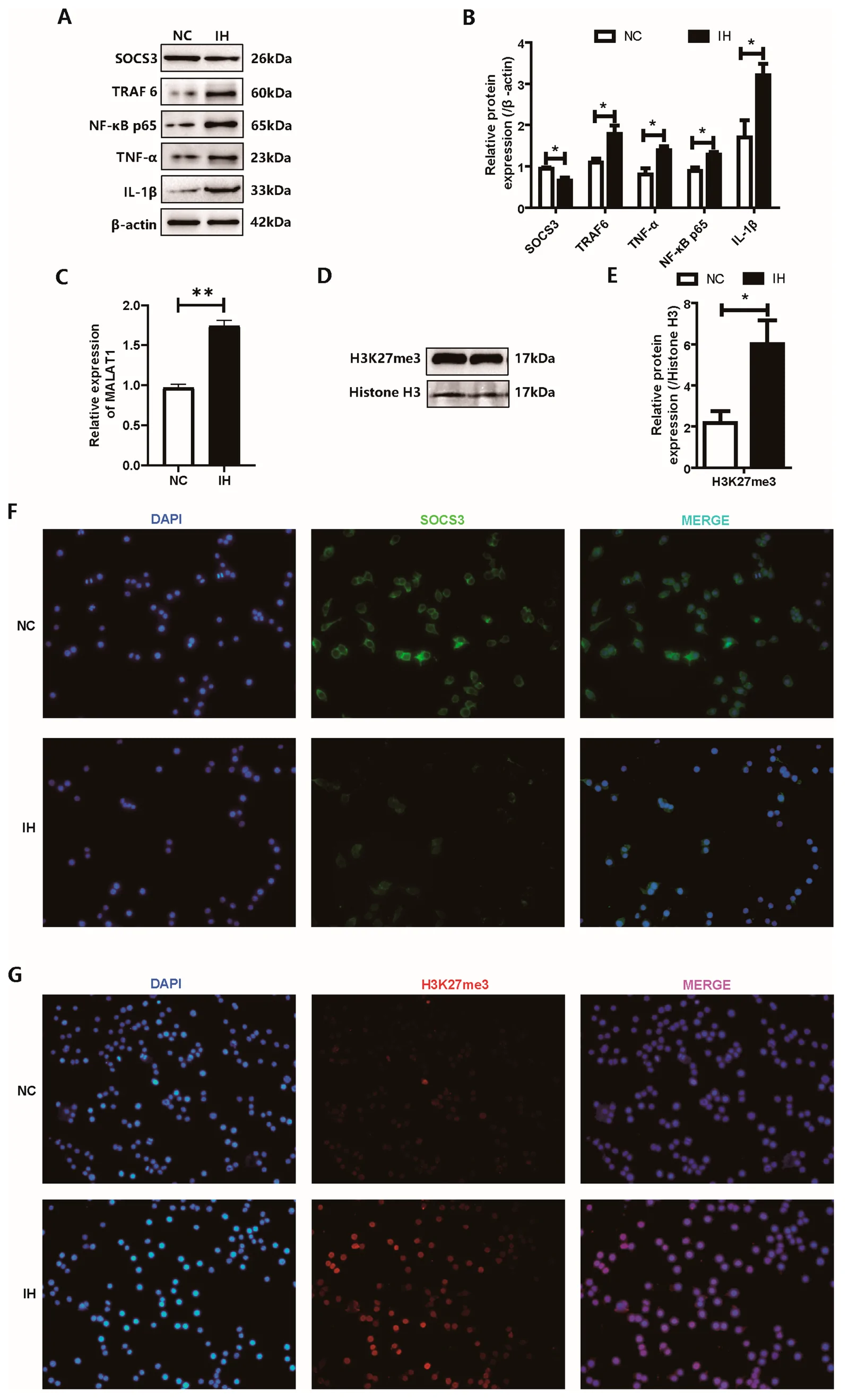
Changes in the expression of SOCS3 and related factors in BV2 microglial cells exposed to intermittent hypoxia (IH). (**A**,**B**) Western blot analysis of SOCS3, TRAF6, NF-κB-p65, TNF-α and IL-1β protein levels normalized to β-actin. (**C**) qRT-PCR analysis of MALAT1 transcript expression. (**D**,**E**) Western blot detection of H3K27me3 protein expression normalized to histone H3. (**F**) Immunofluorescence staining for SOCS3 (green) in BV2 cells. Nuclei were counterstained with DAPI (blue). (**G**) Immunofluorescence staining for H3K27me3 (red) in BV2 cells. Nuclei were counterstained with DAPI (blue). Data are presented as mean ± SEM. *n* = 3 independent biological replicates per group. * *p* < 0.05, ** *p* < 0.01 vs. NC group. *p*-values were calculated using an unpaired Student’s *t*-test.

It is worth noting that the results of our in vitro and in vivo experiments were basically the same. We measured the expression of SOCS3, TRAF6, NF-κB-p65, TNF-α, and IL-1β in the two groups of BV2 cells by WB (Figure 3A,B). The results showed that the expression of TNF-α and IL-1β in the IH group was significantly higher than that in the NC group, which indicated that IH exposure triggered the inflammatory activation of BV2 microglia. In addition, we also found that the expression of SOCS3 was decreased in IH-exposed BV2 cells, while the expression of TRAF6 and NF-κB-p65 was increased. Similarly, the expression of MALAT1 was higher in the IH group (Figure 3C). We measured the expression of H3K27me3 relative to histone H3 in the two groups of BV2 cells by WB, and the results also showed that the H3K27 trimethylation level in the IH group was higher (Figure 3D,E). Finally, we verified the expression of SOCS3 and H3K27me3 in the two groups of BV2 cells by IF analysis (Figure 3F), and the results were consistent with the Western blot results. The expression of SOCS3 in IH-exposed BV2 cells decreased, while H3K27me3 was significantly increased.

### 3.4. SOCS3 Overexpression Was Associated with Lower Inflammatory Protein Levels in IH-Exposed BV2 Cells

**Figure 4:**
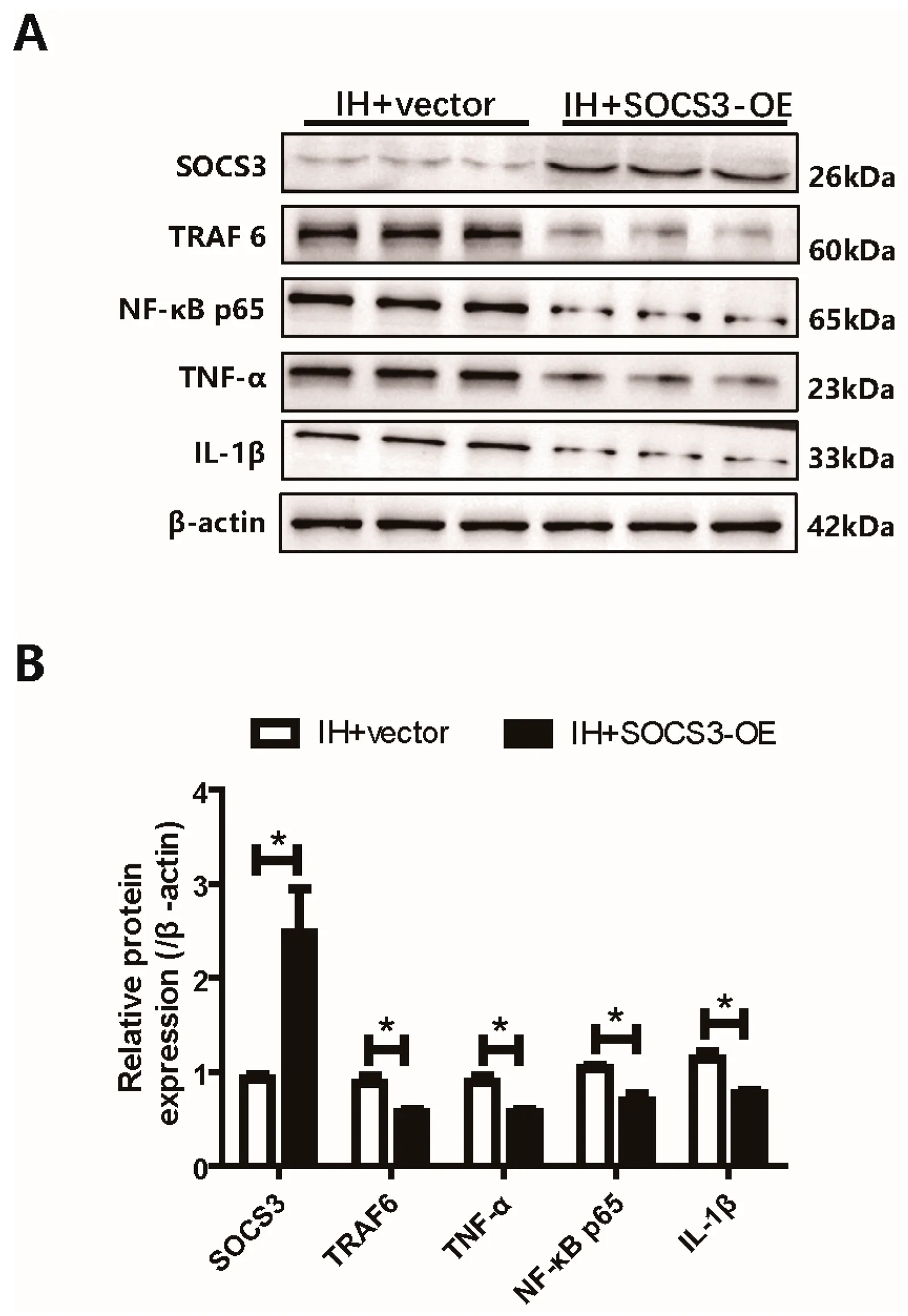
SOCS3 suppresses microglial inflammation via the TRAF6/NF-κB pathway. (**A**,**B**) Western blot analysis of TRAF6, NF-κB-p65, TNF-α and IL-1β protein levels normalized to β-actin after SOCS3 overexpression in BV2 cells. * *p* < 0.05 vs. the IH + Vector group. Data are presented as mean ± SEM, *n* = 3 independent biological replicates per group. *p*-values were calculated using an unpaired Student’s *t*-test.

Our previous results indicated that the downregulation of SOCS3 could promote the inflammatory response of microglia through the TRAF6/NF-κB pathway. To further explore the role of SOCS3 in the inflammatory activation of microglia, we transfected the SOCS3 overexpression plasmid into BV2 cells to increase the expression of SOCS3. Then, after IH exposure, the expression of TRAF6 and NF-κB-p65 and the inflammatory factors TNF-α and IL-1β was measured by WB (Figure 4A,B). The results showed that the expression of TRAF6, NF-κB-p65, TNF-α, and IL-1β in BV2 cells after SOCS3 overexpression was significantly decreased compared with that in the control vector group. These results are consistent with an anti-inflammatory effect of SOCS3 overexpression in BV2 cells. The measurements of total TRAF6 and p65 protein do not, by themselves, establish reduced NF-κB activation or prove pathway mediation.

### 3.5. MALAT1 Knockdown Altered H3K27me3 and SOCS3 Levels in IH-Exposed BV2 Cells

**Figure 5:**
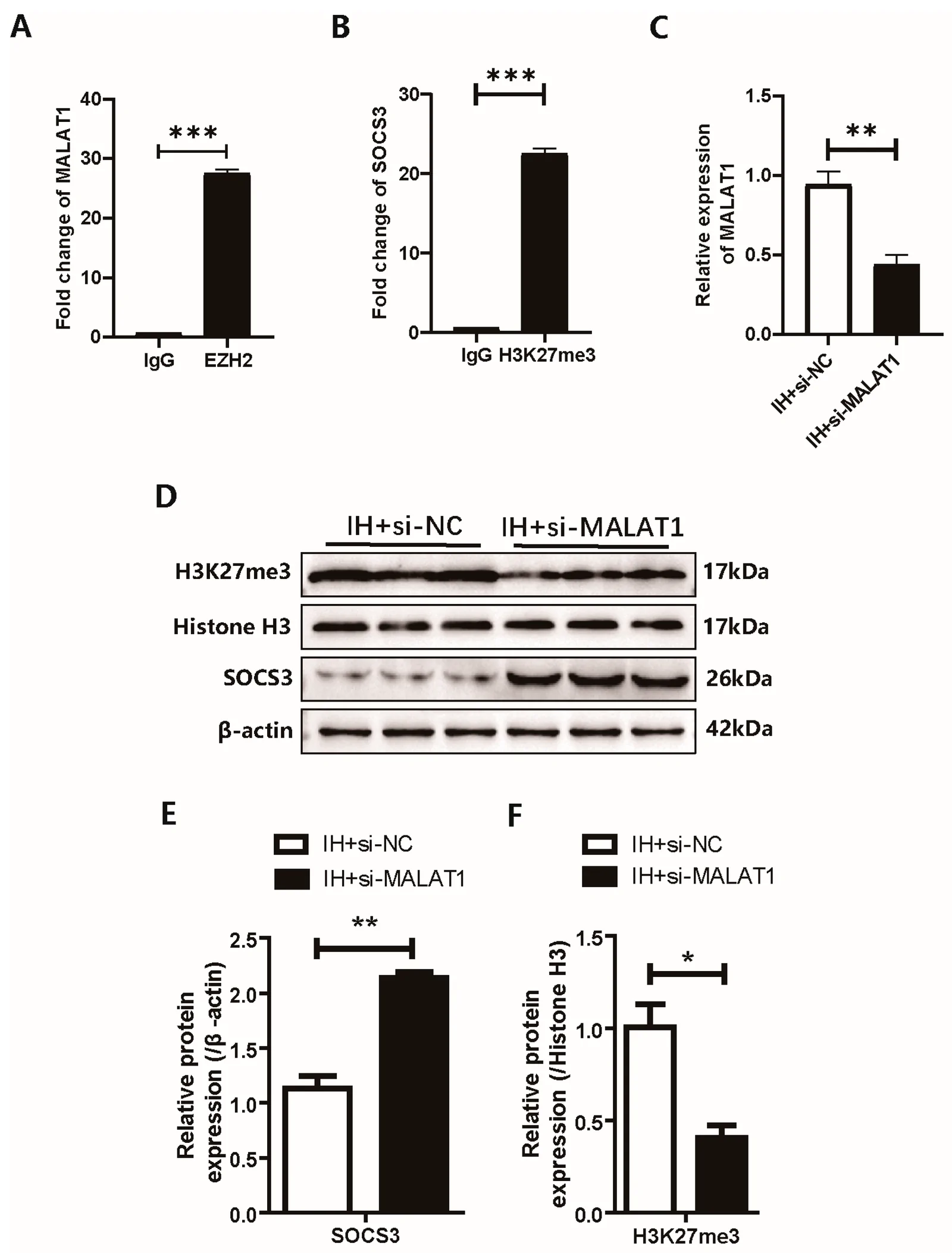
Association among MALAT1, H3K27me3 modification and SOCS3 expression under intermittent hypoxia. (**A**) RIP-qPCR assay validating the physical interaction between MALAT1 and EZH2 in BV2 cells. (**B**) ChIP-qPCR analysis detecting H3K27me3 enrichment at the SOCS3 promoter. (**C**) qRT-PCR confirmation of MALAT1 knockdown efficiency after transfection with MALAT1 siRNA. (**D**–**F**) Western blot detection of H3K27me3 and SOCS3 protein levels following MALAT1 silencing. Data are presented as mean ± SEM. *n* = 3 independent biological replicates per group. * *p* < 0.05, ** *p* < 0.01, *** *p* < 0.001. *p*-values were calculated using an unpaired Student’s *t*-test.

Our previous results showed that both MALAT1 and H3K27me3 were elevated in BV2 cells after IH exposure, so we hypothesized that MALAT1 could promote the trimethylation of H3K27. Since EZH2 can catalyze the trimethylation of H3K27, we used the RIP assay to examine the binding of MALAT1 and EZH2 in BV2 cells (Figure 5A). The results showed that EZH2 could significantly enrich MALAT1. Then, we used a ChIP assay to examine the binding of H3K27me3 to the SOCS3 promoter sequences (Figure 5B). The results showed that H3K27me3 was significantly enriched in the promoter sequences of SOCS3. To further verify the hypothesis that MALAT1 regulates the expression of SOCS3, we transfected BV2 cells with MALAT1 siRNA and performed IH exposure. After verifying that MALAT1 was knocked down (Figure 5C), we measured the expression of H3K27me3 and SOCS3 by WB (Figure 5D–F). The results showed that after reducing the expression of MALAT1, the expression of H3K27me3 also decreased, while the expression of SOCS3 was increased significantly.

**Figure 6:**
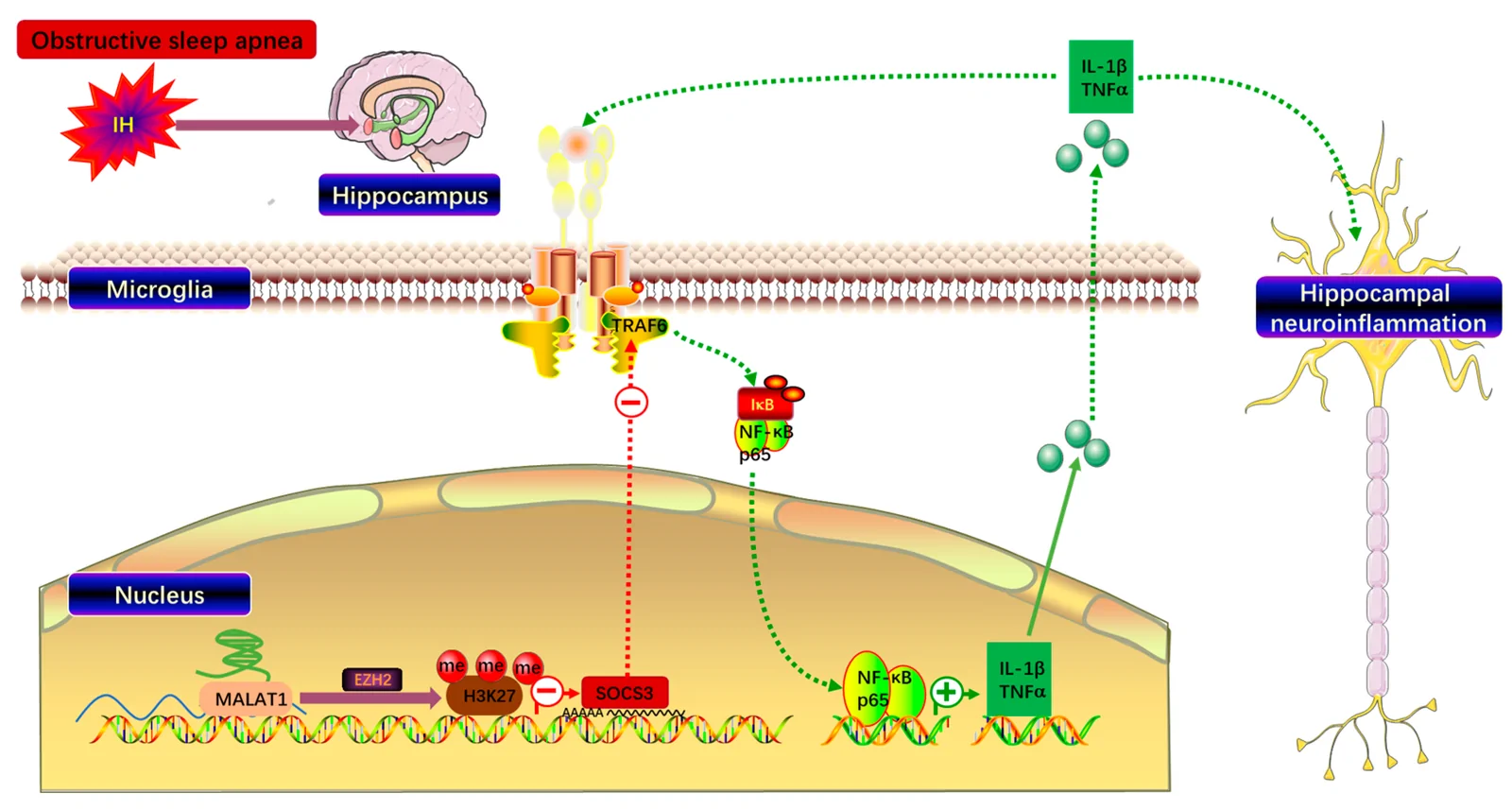
SOCS3 mediates intermittent hypoxia-induced microglia-dependent hippocampal neuroinflammation; targeting the MALAT1/H3K27me3/SOCS3/TRAF6/NF-κB axis offers a potential therapeutic strategy against OSA-associated cognitive deficits. Symbols: minus sign (−) indicates inhibitory effect; plus sign (+) indicates promoting effect.

Together, these findings associate MALAT1 with EZH2 and link MALAT1 knockdown to reduced total H3K27me3 and increased SOCS3 protein in IH-exposed BV2 cells. EZH2 dependence and promoter-specific changes after MALAT1 knockdown remain to be tested (Figure 6).

## 4. Discussion

OSA is the most common type of sleep disorder. The prevalence of moderate-to-severe OSA in the population is very high, with 49.7% of cases occurring in men and 23.4% in women [18]. OSA can cause IH, mild hypercapnia, and wakefulness during sleep. The hippocampus plays a key role in memory, navigation and cognition [19]. As sentinel immune cells in the brain, microglia can recognize harmful stimuli and respond by producing inflammatory cytokines, such as TNF-α, IL-6, IL-1β, interferon-γ (IFN-γ) and several chemokines [20]. There are a large number of IL-1β receptors, TNF-α receptors and high mobility group protein B1 (HMGB1) receptors in the hippocampus associated with learning and memory functions, suggesting that this region is particularly vulnerable to inflammation [21]. There are many clinical imaging studies showing that the increased severity of OSA is related to thinning of the cortex in the molecular layer of the hippocampal dentate gyrus and abnormal connections in the hippocampal network [22,23]. In our study, we used the Morris water maze to test the spatial learning and memory abilities of mice exposed to IH, and the results showed that IH exposure severely impaired the hippocampal functions of mice. Then, we isolated the hippocampus and found that the expression of TNF-α and IL-1β in the hippocampus of mice exposed to IH was significantly increased, and the same results were also found in the in vitro experiments with BV2 cells. These findings demonstrate increased inflammatory markers in whole hippocampal tissue and in BV2 cells following IH. Without cell-specific measurements or intervention in vivo, the source of the hippocampal markers and their causal contribution to behavioral changes cannot be determined.

Since its discovery, SOCS has been characterized as the regulatory cornerstone of intracellular signal transduction. Cytokine signaling regulates various pathophysiological processes [24], including the immune response and inflammation [25]. Although cytokines are needed to control infection, excessive cytokine production may lead to local or systemic pathological reactions. Furthermore, it is necessary to downregulate cytokine signal transduction through the SOCS protein [26]. A recent study showed that the expression of SOCS3 was reduced in a rat model of PD related to neuroinflammation [27]. Interestingly, we also found that the expression of SOCS3 was inhibited in the OSA model. Inflammation is accompanied by the occurrence and development of OSA, and long-term hypoxia can induce the activation of NF-κB-dependent inflammatory pathways [28]. TLRs are receptor proteins that can stimulate the NF-κB pathway and release inflammatory mediators [29]. TRAF6 is one of the key molecules in the TLR signaling pathway, and it is involved in the signal transduction of the tumor necrosis factor receptor and TLR/IL-1R superfamilies and has been shown to regulate cell death, survival, and the cellular response to stress [30]. In addition, TRAF6 knockout can inhibit NF-κB signaling and downregulate TLR-induced cytokine production [31]. Because SOCS3 has E3 ubiquitin ligase activity [32], it can inhibit IL-1 signal transduction by promoting the ubiquitination of TRAF6 [33]. Studies have shown that the upregulation of SOCS3 can inhibit TLR4-induced microglial activation [34,35]. In our study, TRAF6 and total NF-κB p65 protein levels were lower after SOCS3 overexpression in BV2 cells. Activation and mediation of NF-κB signaling were not directly demonstrated.

The posttranslational modifications of the N-terminal tails of core histones include methylation, acetylation, ubiquitination and phosphorylation, and these modifications can affect the chromatin configuration to regulate the accessibility of transcription factors and the transcriptional activity of nearby genes [36]. Studies have shown that the trimethylation of H3K27 is related to gene expression inhibition [37]. In addition, the trimethylation of H3K27 can lead to increased production of proinflammatory cytokines induced by bacteria [38]. In our study, we also found increased expression of H3K27me3 in the hippocampus of IH-exposed mice and BV2 cells. Therefore, we hypothesized that H3K27me3 might be related to the downregulation of SOCS3 expression and verified the interaction of H3K27me3 with the SOCS3 promoter region.

PRC2 is responsible for the methylation of lysine 27, and it contains four core subunits, EZH2 or EZH1, suppressor of zeste (SUZ) 12, embryonic ectoderm development (EED) and retinoblastoma-binding protein 7 (RBBP7) or RBBP4, in which the catalytic subunit is the EZH2 protein containing the SET domain [39]. EZH2 is involved in many diseases associated with the abnormal expression of H3K27me3 [40]. After EZH2 trimethylates H3K27, EED binds to H3K27me3 through its WD40 domain, resulting in a conformational change and thereby enhancing its catalytic activity, which is called allosteric activation [41]. By binding its own product via EED, PRC2 can spread to neighboring nucleosomes to deposit H3K27me3, and H3K27me3 can also serve as the recognition site of EED, thus forming a positive feedback loop to maintain inflammatory responses [42].

As an lncRNA, MALAT1 can regulate neuronal apoptosis and participate in the pathogenesis of various neurological diseases [43,44,45]. Previous studies by our research group have demonstrated that the increased expression of MALAT1 in microglia is increased after IH exposure and can participate in the inflammatory response through the miR-224-5p/NLRP3/IL-1β pathway [17]. Interestingly, in addition to sponging miRNAs, cis-acting lncRNAs can also mediate and stabilize the effect of PRC2 on chromatin. A study showed that MALAT1 accelerates skeletal muscle cell apoptosis and the inflammatory response in sepsis by recruiting EZH2 [46]. Therefore, we hypothesize that MALAT1 can also promote the trimethylation of H3K27 to downregulate SOCS3 expression by recruiting EZH2. RIP showed an association between EZH2 and MALAT1 in BV2 cells; it did not demonstrate recruitment of EZH2 to the SOCS3 promoter. By knocking down the expression of MALAT1 in BV2 cells, we found that the expression of H3K27me3 decreased, while the expression of SOCS3 increased significantly. These findings support the proposed relationship but do not establish that EZH2 is required for the MALAT1-dependent change in SOCS3.

In summary, IH was associated with lower SOCS3 and higher inflammatory marker levels in whole hippocampal tissue and BV2 cells. SOCS3 overexpression reduced inflammatory protein levels in BV2 cells. Our RIP, ChIP-qPCR, and MALAT1 knockdown results are compatible with a possible MALAT1/EZH2/H3K27me3 relationship, but they do not establish EZH2-dependent repression of SOCS3. We did not identify the in vivo cellular source of the inflammatory markers or test whether this pathway mediates water maze performance. Cognitive assessment was limited to the Morris water maze. These questions require cell-specific and causal experiments.

## Data Availability

All data supporting the findings of this study are included within the manuscript. No additional publicly archived datasets were generated in this work.
